# Genome-wide prediction and functional analysis of *WOX* genes in blueberry

**DOI:** 10.1186/s12864-024-10356-5

**Published:** 2024-05-02

**Authors:** Yanwen Wang, Lei Yang, Wenzhu Geng, Rui Cheng, Hongxia Zhang, Houjun Zhou

**Affiliations:** 1 The Engineering Research Institute of Agriculture and Forestry, Ludong University, Yantai, 264025 Shandong China; 2 Bestplant (Shandong) Stem Cell Engineering Co., Ltd, 300 Changjiang Road, Yantai, 264001 Shandong China

**Keywords:** Blueberry, Transcription factor, Bioinformatics, Tissue-specific expression

## Abstract

**Background:**

*WOX* genes are a class of plant-specific transcription factors. The WUSCHEL-related homeobox (WOX) family is a member of the homeobox transcription factor superfamily. Previous studies have shown that WOX members play important roles in plant growth and development. However, studies of the WOX gene family in blueberry plants have not been reported.

**Results:**

In order to understand the biological function of the *WOX* gene family in blueberries, bioinformatics were used methods to identify *WOX* gene family members in the blueberry genome, and analyzed the basic physical and chemical properties, gene structure, gene motifs, promoter cis-acting elements, chromosome location, evolutionary relationships, expression pattern of these family members and predicted their functions. Finally, 12 genes containing the WOX domain were identified and found to be distributed on eight chromosomes. Phylogenetic tree analysis showed that the blueberry *WOX* gene family had three major branches: ancient branch, middle branch, and WUS branch. Blueberry *WOX* gene family protein sequences differ in amino acid number, molecular weight, isoelectric point and hydrophobicity. Predictive analysis of promoter cis-acting elements showed that the promoters of the *VdWOX* genes contained abundant light response, hormone, and stress response elements. The *VdWOX* genes were induced to express in both stems and leaves in response to salt and drought stress.

**Conclusions:**

Our results provided comprehensive characteristics of the *WOX* gene family and important clues for further exploration of its role in the growth, development and resistance to various stress in blueberry plants.

**Supplementary Information:**

The online version contains supplementary material available at 10.1186/s12864-024-10356-5.

## Background

The WOX protein belongs to the homeobox (HOX) superfamily [1], and is composed of 65 amino acids, which fold into a DNA-binding domain with three helixes in space [2]. Homeodomain is characterized by a highly conserved “helix-loop-helix-turn-helix, with all WOX members containing a homologous domain of 60–66 amino acid residues [3]. The sequence specificity of the domain distinguishes the *WOX* gene family from other homeobox. The homology domain binds to DNA via a helix-turn-helix (HTH) structure. The characteristic of the HTH motif is that two α-helices tightly connected to DNA and linked through a loop [4].

In plants, members of the *WOX* gene family play important roles in developmental processes, including embryonic development, maintenance of meristematic tissue stem cells, development of lateral organs, seed formation, and regeneration of detached tissues and organs [5]. The *WOX* gene family has been identified in many plants, such as maize [6], apple [7], rice [8] and soybean [5], including 15 *WOX* genes in *Arabidopsis*. Based on their evolutionary relationships, they are classified into three branches, the WUS branch (WUS Clade), Intermediate Clade and Ancient Clade [3, 4]. The members of the blueberry *WOX* gene family, like those of the *Arabidopsis WOX* gene family, are distributed among three clades. The WUS Clade promotes stem cell proliferation in floral meristems, the Intermediate Clade demonstrates effective expression during embryonic development and cell division processes, and the Ancient Clade functions in root development and floral transition [5]. Several members of the *Arabidopsis* WOX family have been shown to be important for maintaining embryonic patterns in stem cells, stem apical meristem (SAM), root apical meristem and organs formation [9]. The AtWUS proteins are involved in maintaining the homeostasis of stem cells in SAM at all developmental stages, and the homeostasis of SAM is disrupted in *Arabidopsis wus* mutants [10]. Similarly, VdWOX3, belonging to the WUS Clade, exhibits the highest expression level in stems, indicating its potential significant role in stem development. The *AtWOX2* protein is expressed in germ cells and is required for embryonic tip development [11]. *AtWOX3* is expressed in the leaf primordium and floral organ margins, and forms the peripheral histological structures of nutrient and floral organs [12]. In rice (*Oryza sativa*), *OsWOX3* is involved in organ development, growth of leaf lateral axes and vascular formation, morphogenesis of spikelet lemma and palea, and development of tillers and lateral roots [13, 14]. *AtWOX4* plays a crucial role in the regulation of growth hormone in the formation layer [15]. *AtWOX5* is necessary for the maintaining the homeostasis mechanism of IAA in root tip meristem tissues [16]. *AtWOX6* is a homologous domain protein encoded by the *PRETTY FEW SEEDS2* gene, which plays a regulatory role in ovule development [17]. *AtWOX6* (also known as *HOS9-1*) plays a role in the physiological processes of plant resistance to freezing by affecting gene activity independent of the CBF pathway [18]. *AtWOX7* regulates the development of plant lateral roots as well as the distribution of sugars [19]. In blueberries, *VdWOX9* exhibits higher expression levels in roots compared to other tissues, which may suggest its involvement in regulating root development, given its closer phylogenetic relationship with *AtWOX7* in the evolutionary tree. *AtWOX8* regulates growth hormone gradient formation during early embryonic development [20]. *STIMPY* (*STIP*; also known as *WOX9*) may play a role in organisation by maintaining cell division and preventing premature differentiation, and *STIP* identifies a new genetic pathway that combines developmental signaling with cell cycle control [21]. Moreover in wheat, *TaWOX9* promotes root development [22].

In *Arabidopsis*, *WOX11* associates upstream growth hormone signaling with downstream transformation of cell morphology to initiate the formation of root and healing tissue primordia [23]. Several studies have found that the switch in expression from *WOX11/12* to *WOX5/7* is crucial for the formation of root primordia during new root organogenesis in plants [24]. *WOX11* and *WOX12* are involved in the life cycle of the most primitive cells that form root organs in *Arabidopsis* [25]. *AtWOX13* is involved in initiation and development of primordial and lateral root, and is dynamically expressed during pistil and embryonic development. The *WOX13* promotes replicon development by blocking the activity of *JAG/FIL* genes in the inner tissues [26, 27]. In contrast, overexpression of *WOX14* stimulates the expression of *GA3ox* anabolic genes and represses *GA2ox* catabolic genes, which promotes the accumulation of bioactive GA. *WOX14* promotes the differentiation and lignification of vascular cells in the inflorescence stems of *Arabidopsis* [28].

*WOXs* gene not only play a role in plant development, but also in response to abiotic stresses. *AtWOX6* (also known as *HOS9-1*) plays a role in the physiological processes of plant resistance to freezing by affecting gene activity independent of the CBF pathway [18]. Mutant alleles of *AtWOX6* are expressed in response to freezing stress [18]. In rice, most *WOXs* gene respond to abiotic stress stimuli such as drought, salt and cold [8]. In blueberries, after salt treatment, the expression levels of *VdWOX4* and *VdWOX11* are upregulated. Following drought treatment, the expression levels of *VdWOX4* and *VdWOX8* are upregulated, while those of *VdWOX7* and *VdWOX9* are downregulated. These phenomena collectively suggest the response of the blueberry *WOX* family to abiotic stressors. In woody plants grapes, the *VvWOX* genes is a key regulator of somatic embryogenesis in grapes [29]. In the gymnosperm European spruce, it contains a number of *WOX* related genes, many of which are expressed during embryonic development [30]. Interestingly, this study revealed through phylogenetic tree and collinearity analysis that blueberries share a closer evolutionary relationship with grapes, both being woody plants. Gene structure analysis further revealed that the number of introns ranges from 1 to 3. Similar results were also observed in leguminous plant species.

In summary, WOX transcription factors mainly affect plant growth and development by altering the expression of downstream genes. It is noteworthy that the cloning and functional studies of WOX proteins mainly focus on model plants such as *Arabidopsis* and rice. However, no comprehensive molecular evolutionary studies have been conducted on blueberry (evergreen blueberry: *Vaccinium darrowii*). Blueberry (*Vaccinium darrowii*) is one of the top five health foods for human beings and is recognised as the “King of Fruits of the World”, and given the current status of blueberries as a bioactive ingredient and valuable food, they have greater nutritional and health potential [31].

In this study, we comprehensively identified and analyzed the structure, motifs, cis elements and homology characteristics of the *WOX* gene family in blueberry. Twelve *WOX* genes were identified from the entire genome of sequenced evergreen blueberry, and their functions were predicted through phylogenetic analysis and quantitative analysis of different tissues, This study will provide further insights into the evolution and functional characteristics of the *WOX* gene family in blueberry and also provides evidence for further research on the role of the WOX family in growth, development and stress resistance. To provide genetic resources and scientific basis for breeding high-yielding, high-quality, and stress-resistant blueberry cultivars.

## Materials and methods

### Identification of blueberry *VdWOX* gene family members

To identify the *WOX* family in the blueberry genome, we employed two distinct methodologies. Firstly, The nucleotide and protein sequences of blueberry were downloaded from the NCBI database (https://www.ncbi.nlm.nih.gov/), and the WOX family protein homeodomain feature file was downloaded from the website Pfam (https://pfam.xfam.org/) (PF00046), built a Hidden Markov Model using HMMER 3.0, and searched for proteins containing homeodomain in the above mentioned database (NCBI) using the hmm search programme in HMMER 3.0, set the E-value threshold to 0.01, and redundant protein sequences were manually removed. Secondly, a local BLASTp was conducted against the blueberry genome database using 15 known Arabidopsis sequences to search for WOX protein sequences, with an E-value threshold set at 1 × 10 − 5. A summary of the results from both methods was provided, and redundant sequences were eliminated, resulting in a total of 15 protein sequences identified as candidate WOX family proteins in blueberries. Their conserved domains were validated using NCBI-CDD (https://www.ncbi.nlm.nih.gov/cdd/). The ExPASy online website (https://web.expasy.org/protparam/) was used to predict the physicochemical properties of the *VdWOX* gene family, including the number of amino acids, molecular weight, isoelectric point, hydrophilicity and the subcellular localization of VdWOXprotein was predicted using the website (http://www.csbio.sjtu.end.cn/bioinf/Cell-PLoc-2/). Chromosome location, sequence alignment, gene structure, conserved motifs and three‑dimensional domain analysis.

The chromosomal location and gene structure of *VdWOX* genes were visualized using TB tools based on information from the GFF annotation file (NCBI) [32]. WOX protein sequence comparison was performed using DNAMAN. Conserved motifs were analyzed using the online website MEME (https://meme-suite.org/meme/). Perform secondary structure analysis of blueberry *WOX* gene family proteins using SOPMA (https://npsa-prabi.ibcp.fr/cgi-bin/npsa_automat.pl?page=npsa_sopma.html). The VdWOX protein sequence were analyzed for three dimensional domain using the online tool SWISS-MODEL (https://swissmodel.expasy.org/). To ensure the accuracy of the model, AtWOX protein with high similarity to VdWOX protein were used as templates.

### Evolutionary tree analysis of different species of WOXs

To construct the phylogenetic tree of WOXs, the protein sequences of WOXs from *Arabidopsis thaliana* [33] and maize [34] were extracted from the previous study, and the protein sequences of WOXs from grape and barley were downloaded from the PlantTFDB (http://planttfdb.gao-lab.org/) database. Phylogenetic trees were constructed by the method of nearest-neighbour joining (NJ) after multiple sequence comparison using MEGA X software with a self-expansion value of 1000 [35]. The relevant species protein sequences are listed in Table S1.

### Synteny relationship analysis

Blueberry genome annotation files (GFF files) were downloaded from the NCBI database. The Multiple Covariance Scanning Toolkit from TBtools was used to visualize the covariance relationships of WOXs from different species.

### Identification of cis‑acting elements in VdWOX promoter

The 2 kb upstream of each *VdWOX* genes was obtained from the NCBI database as promoter region. The cis-acting elements were predicted on Plant-CARE website (http://bioinformatics.psb.ugent.be/webtools/plantcare/html/) and analyzed using Excel for classification [36].

### Plant materials, growth conditions and stress treatments

In this study, O’Neill blueberry seedlings were used as the experimental material. One year old blueberry seedlings were grown in a plant growth chamber with a photoperiod of 16 h of light and 8 h of darkness, a light intensity of 100 µmolm^-2^s^-1^ at 23℃ (light) and 20℃ (dark). Different tissue parts such as roots, stems, leaves, flowers, young fruits as well as mature fruits of normal growing blueberries were taken. Blueberry seedlings were treated with 200 mM NaCl for 0 d, 1 d, 3 d, 5 d, 7 d and 9 d, and were treated with drought for 1 d, 3 d, 6 d, 9 d, 12 d and 15 d. After treatment, the tissues of the plants were taken and total RNA was extracted.

### RNA isolation and quantitative real‑time PCR (qRT‑PCR)

Total RNA was extracted using FastPure Universal Plant Total RNA Isolation Kit and cDNA strands were synthesised using HiScript III 1st Strand cDNA Synthesis Kit (Vazyme). *VdTub2* was used as a control, and quantitative PCR was performed with a CFX Connect Real-Time System (Bio-Rad, America) using the ChamQ Universal SYBR qPCR Master Mix (Vazyme). *VdWOX* genes specific primers were designed using Premier 5.0 software, and the sequences are listed in Table S2. Reaction conditions: pre-denaturation: 1 min at 95 °C, denaturation: 15 S at 95 °C, annealing extension: 15 S at 60 °C and cycling 40 times. The method of comparing CT values was used to assess the relative expression level of qRT-PCR products [37].

### Statistical analysis

All data were analyzed with Origin 8 and SPSS19. Two-tailed Student’s t test was used for statistical analysis.

## Results

### Identification of blueberry *WOX* gene family members

To understand the possible biological functions of WOX protein in blueberries, we searched the hidden Markov model (HMM) of homeodomain. After the selection of conserved domains in the potential candidate proteins, a total number of 12 *WOX* encoding genes were identified in the blueberries genome. Based on their chromosomal locations, they were designated as *VdWOX1*- *VdWOX12*, respectively (Table 1). The amino acid numbers of various proteins encoded in the blueberry *WOX* gene family range from 171 to 447. The molecular weight range was 19839.3 (*VdWOX9*) − 48547.2 (*VdWOX4*). The isoelectric point was within the range of 5.46 (*VdWOX6*) − 9.92 (*VdWOX11*). The instability index range was 44.85 (*VdWOX5*) − 68.93 (*VdWOX12*), and the average hydrophilicity coefficients were all negative, indicating that they were all hydrophilic proteins. Subcellular localization prediction indicates that all VdWOX proteins are localized in the nucleus.

**Table 1:** Physical and chemical properties of *WOX* gene and its encoding protein

Gene name	Gene ID	Protein size(aa)	Molecular weight	pI	Instability index	GRAVY	Localization prediction
*VdWOX1*	Vadar_g32306	244	27279.05	5.7	62.72	-0.771	Nucleus
*VdWOX2*	Vadar_g43364	328	37118.7	8.44	66.02	-0.775	Nucleus
*VdWOX3*	Vadar_g26736	222	25331.8	8.92	49.02	-0.917	Nucleus
*VdWOX4*	Vadar_g40114	447	48547.2	7.36	57.67	-0.449	Nucleus
*VdWOX5*	Vadar_g23772	229	25742.2	9.35	44.85	-0.844	Nucleus
*VdWOX6*	Vadar_g26031	277	31,454	5.46	56.36	-0.938	Nucleus
*VdWOX7*	Vadar_g26188	313	34283.4	5.89	51.06	-0.812	Nucleus
*VdWOX8*	Vadar_g36824	333	37711.9	9.53	49.64	-0.668	Nucleus
*VdWOX9*	Vadar_g16964	171	19839.3	8.91	63.13	-0.785	Nucleus
*VdWOX10*	Vadar_g6244	207	23,883	9.54	61.99	-0.875	Nucleus
*VdWOX11*	Vadar_g6282	239	26513.7	9.92	53.85	-0.592	Nucleus
*VdWOX12*	Vadar_g6283	219	24441.5	9.38	68.93	-0.748	Nucleus

### Chromosomal localization, developmental tree and multiple sequence comparison analysis of *VdWOX* gene family

**Fig. 1:**
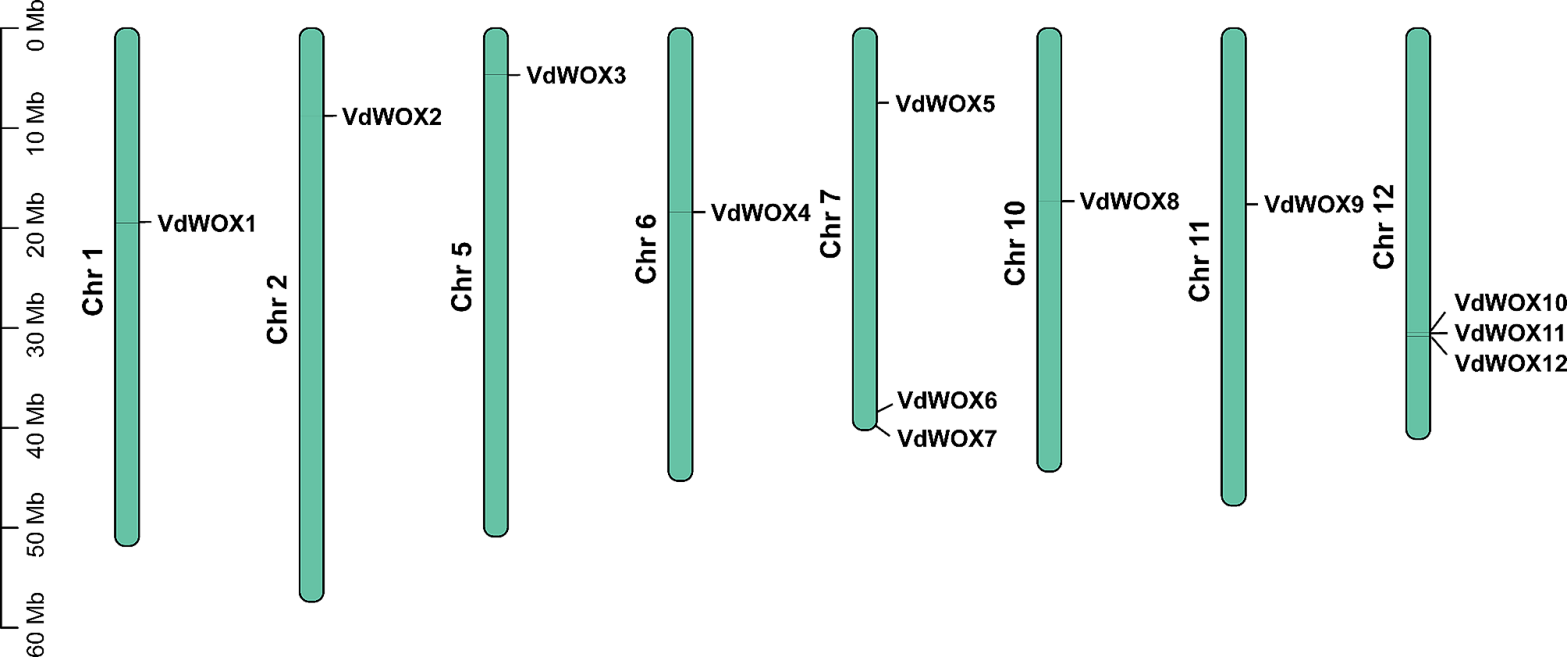
Chromosome distribution of *WOX* gene family members in blueberry. The left axis displays the length of each chromosome, estimated in megabases (Mb)

According to genetic mapping information, the 12 *WOX* gene family members in blueberries were located on 8 chromosomes (Fig. 1). Among them, there were no *WOX* gene family members on chromosomes 3, 4, 8 and 9. There was only one member of the *WOX* gene family on chromosomes 1, 2, 5, 6, 10 and 11. And chromosomes 7 and 12 each had three members of the *WOX* gene family.

**Fig. 2:**
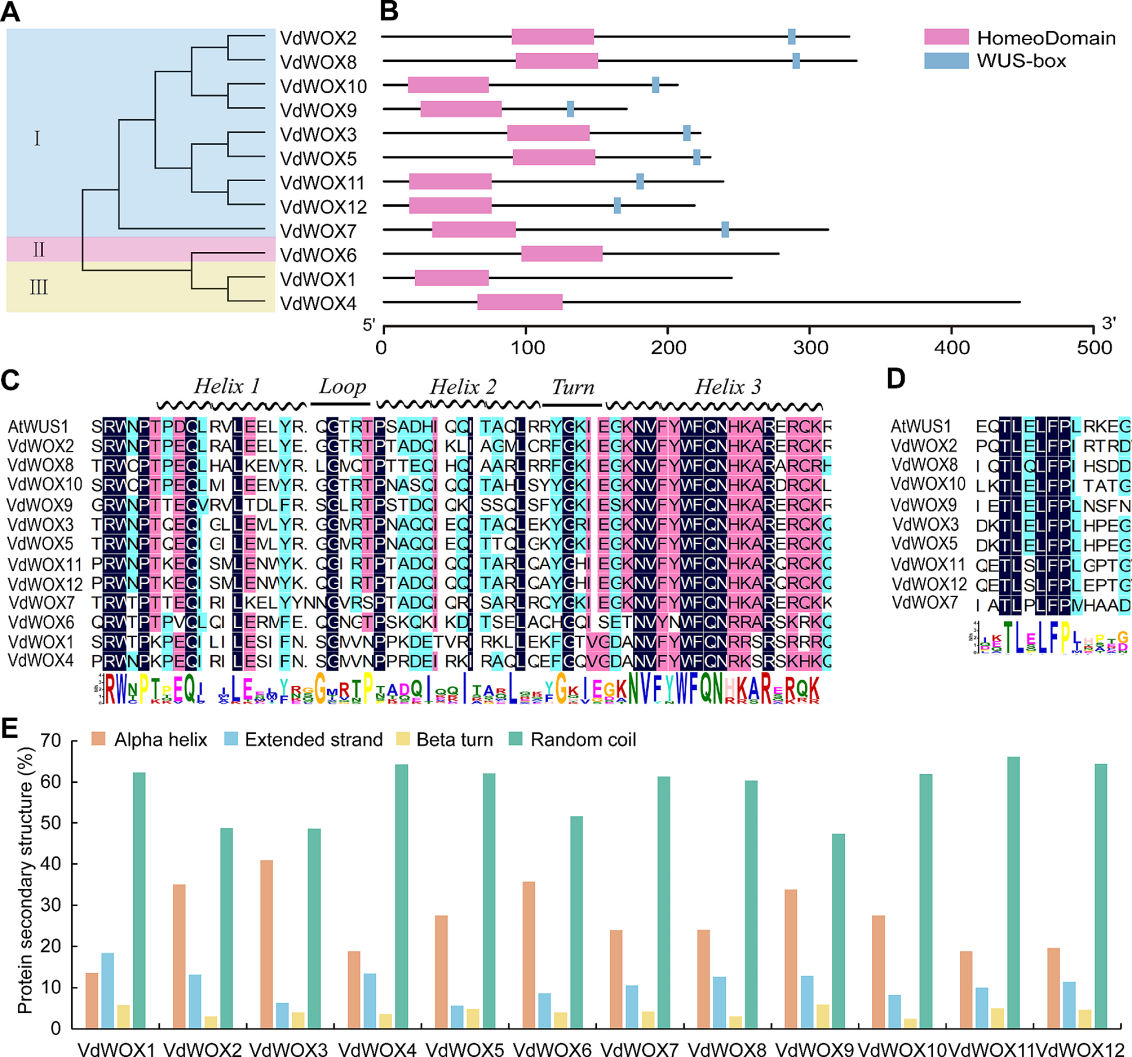
Sequence ratios of blueberry *WOX* gene family proteins for conserved structural domain analysis. (**A**) Phylogenetic tree analysis of WOX proteins in blueberry, I: WUS Clade, II: Ancient Clade, III: Intermediate Clade. (**B**) Domains characterised, light purple represents homeodomain, light blue represents Wus-box, sequence size (bp) is indicated by the scale at the bottom of the figure. (**C**) Sequence of VdWOX proteins homeodomain. (**D**) WUS-box sequence of *VdWOX* gene family proteins. (**E**) Secondary structure statistics of VdWOX proteins sequence

In order to better reveal the functional domains in the *VdWOX* gene family, we performed a developmental tree analysis of 12 VdWOX proteins sequences (Fig. 2A), and classified the VdWOX family proteins into three subfamilies (WUS Clade, Ancient Clade and Intermediate Clade). Meanwhile, we performed a simple modular structure study of the VdWOX proteins sequences using (SMART) (Fig. 2B) and found that all the proteins contained the structural feature of homeodomain, while the members of the WUS Clade subfamily contained the WUS-box motif, highlighting the defining characteristics of the *VdWOX* gene family.

Sequence comparison showed that the distribution of amino acids in the conserved homeodomain of the 12 VdWOX proteins was extremely similar (Fig. 2C). Previous studies reported the presence of 11 conserved amino acids in the homeodomain region, including Q, L and Y in Helix 1 and I, V, W, F, N, K and R in Helix 3 [31]. These amino acids are also conserved in the homeodomain of VdWOX proteins. The conserved amino acid residues in Helix 2 were also found to be P, I and L, as well as F and Q in Helix 3. Proteins in the WUS Clade subfamily were found to contain WUS-box motifs with the sequence “TLXLFP” (where X represents any amino acid) (Fig. 2D). Simultaneously. The secondary structure of WOX family proteins in blueberry was analyzed by SOPMA. The results indicate that the secondary structure of these 12 blueberry WOX family proteins comprise four structural forms: Alpha helix, Extended strand, Beta turn and Random coil. Among these, Random coil constitutes the majority, exceeding 47% in all cases, while Beta turn is the least abundant, ranging between 3% and 6% (Fig. 2E).

### Phylogenetic analysis of the *WOX* gene family

**Fig. 3:**
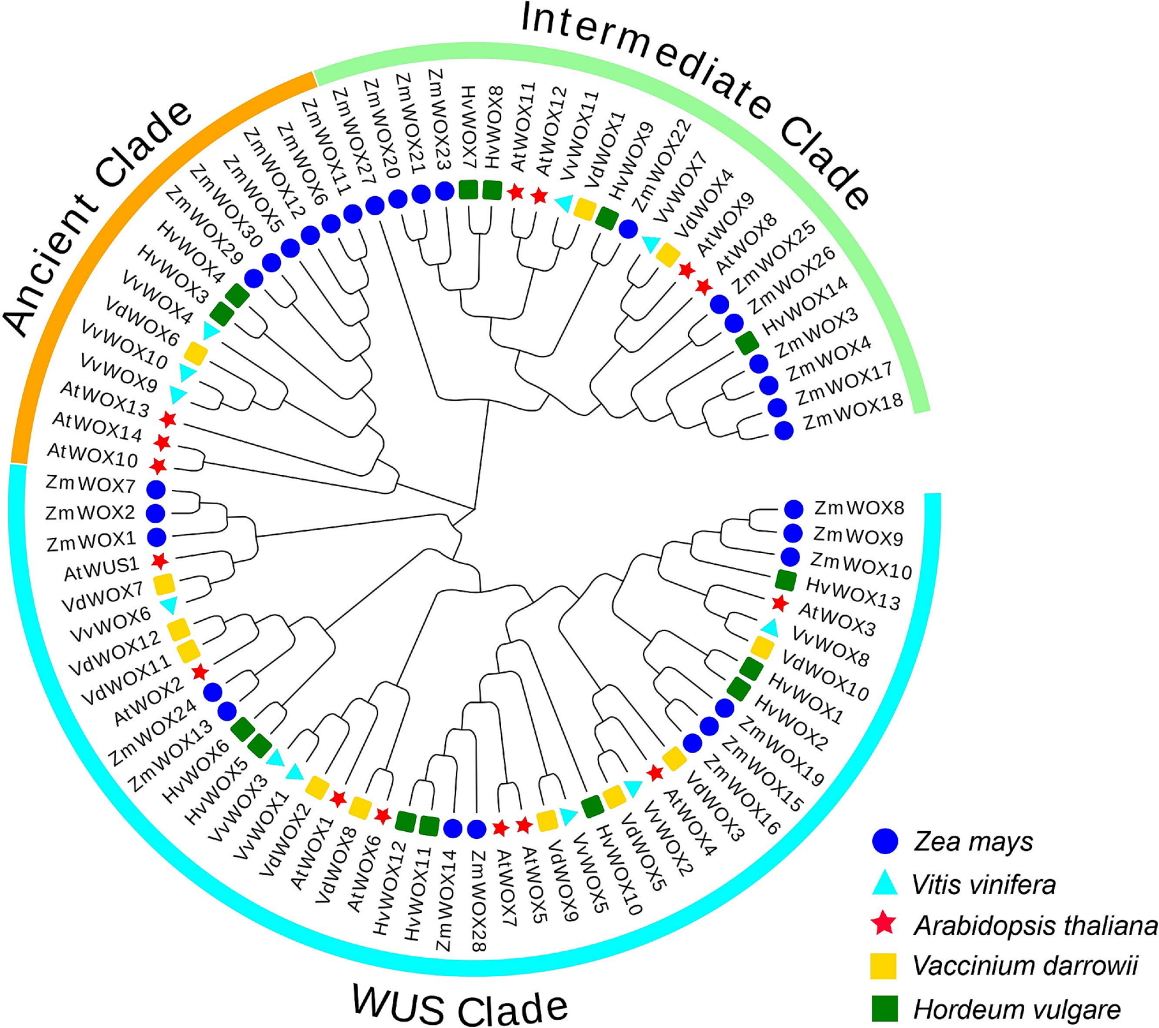
Phylogenetic tree analysis of WOX proteins in *Arabidopsis thaliana*, *Zea mays, Vitis vinifera, Hordeum vulgare* and *Vaccinium darrowii*

To investigate the phylogenetic relationship of *WOX* genes, a total of 82 WOX protein sequences from *Arabidopsis*, maize (*Zea mays*), grape (*Vitis vinifera*), barley (*Hordeum vulgare*) and blueberry (*Vaccinium darrowii*) were used for constructing a phylogenetic tree (Fig. 3). A phylogenetic tree was constructed for the WOX family of five species. The results indicated that members of the *WOX* gene family were divided into WUS Clade, Ancient Clade, and Intermediate Clade, and the number of members of the WUS Clade was larger than that of the Ancient Clade and the Intermediate Clade. There were 44 members in the WUS branch, including 8 in *Arabidopsis*, 13 in maize, 6 in grape, 8 in barley and 9 in blueberry. There were 15 members in the ancient branch, including 3 in *Arabidopsis*, 6 in maize, 3 in grape, 2 in barley and 1 in blueberry. And there were 23 members in the Intermediate branch, including 4 in *Arabidopsis*, 11 in maize, 2 in grape, 4 in barley, and 2 in blueberry. The number of *WOX* family members varied greatly among different species in different evolutionary branches, suggesting that each species underwent different evolutions after differentiation, and the number of genes changed with the evolution of the gene family.

In addition, the homology relationships among the *WOX* gene family of *Arabidopsis*, maize, grape, barley and blueberry were also investigated (Fig. 3). It was found that members of maize, barley and *Arabidopsis* as herbaceous plants appeared to cluster with each other, such as *AtWOX11* and *AtWOX12* in *Arabidopsis* at the Intermediate Clade, *HvWOX7* and *HvWOX8* in barley, *ZmWOX21* and *ZmWOX23* in maize. Similarly, clustering phenomena have also been observed in Nelumbo nucifera, such as *NnWOX6* and *NnWOX9*, *NnWOX1* and *NnWOX4* [38]. Previous studies have found that the relationship between *GmWOXs* and *PvWOXs* is closer, possibly due to the fact that lotus root and soybean both belong to the legume family [5]. The same phenomenon occurred with members of grapes and blueberries as woody plants, such as *VdWOX6* and *VvWOX4* in the ancient Clade and *VdWOX5* and *VvWOX2* in the WUS Clade. This indicated that blueberry and grape were closely related as woody plants. The protein IDs of the required species are shown in Table S1.

### Gene structure and motif analysis of *WOX* gene family in blueberry

**Fig. 4:**
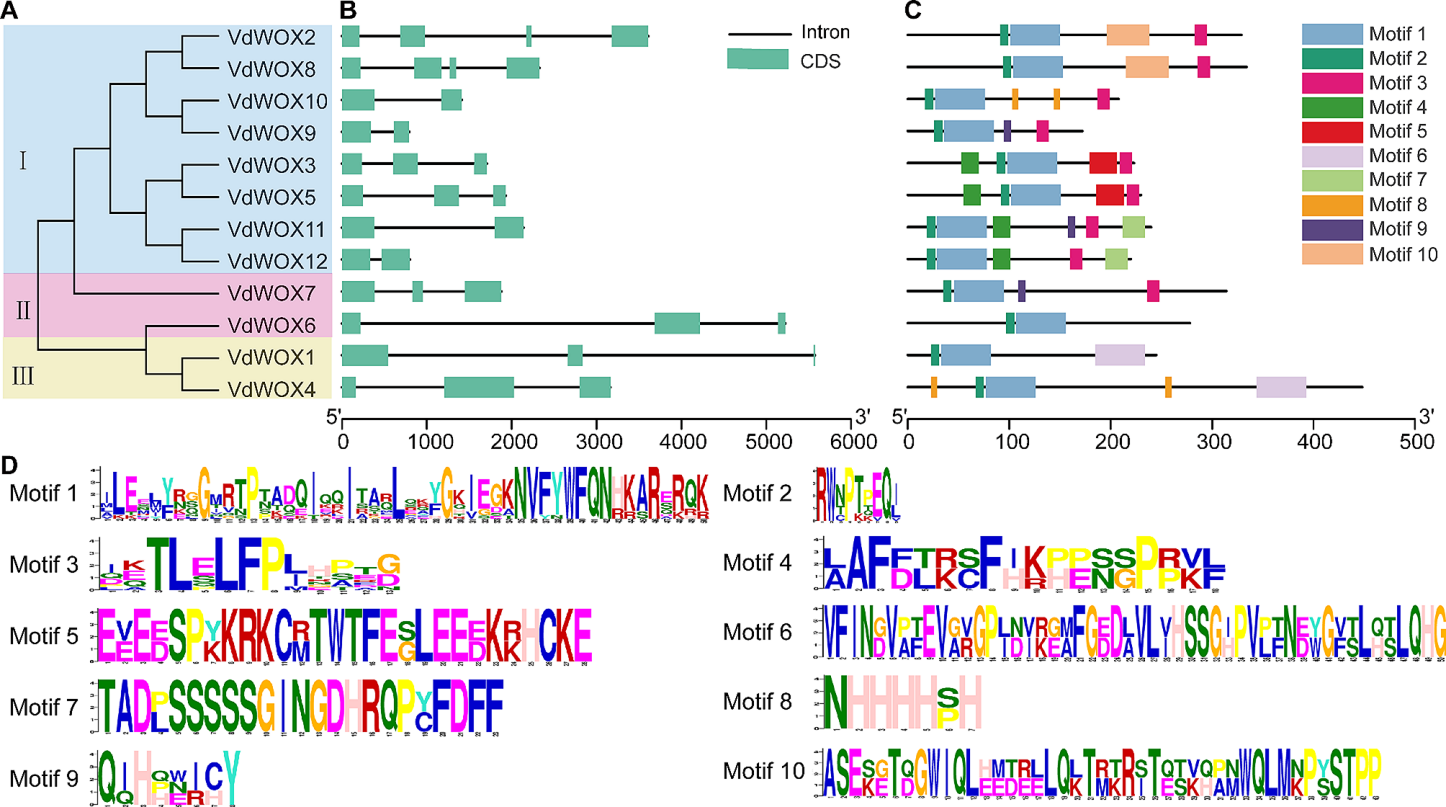
Conservative motif and gene structure analysis of VdWOXs. (**A**) The phylogenetic tree of VdWOXs, I: WUS Clade; II: Ancient Clade; III: Intermediate Clade. (**B**) CDS and intron structures, CDS and introns are represented by light green boxes and black lines, respectively. The sizes of their sequences (bp) are shown with a scale at the bottom of the figure. (**C**) Identification of the conserved motifs in VdWOXs. Boxes in different colors represent different motifs. The sizes of their sequences (aa) are shown with a scale at the bottom of the figure. (**D**) *VdWOX* gene family motif sequences

To investigate the structural diversity of *VdWOX* genes, the distribution of exons and introns was analyzed. Gene structure analysis revealed that the number of introns among members of the *VdWOX* gene family ranges from 1 to 3, a phenomenon also observed in lotus and soybean [5, 38]. Specifically, *SVdWOX9*, *VdWOX10*, *VdWOX11* and *VdWOX12* contain two exons, *VdWOX2* and *VdWOX8* contain four exons, while the remaining six members contain three exons each (Fig. 4). Moreover, to reveal the correlations of *WOX* gene family, their conservative motifs were predicted. Conservative motifs analysis showed that all VdWOX family proteins contained motif 1 and motif 2, and motif 1 appeared before motif 2 in all cases. In the WUS Clade subfamily, WOX proteins all contain motif 3, and in the Ancient Clade subfamily *VdWOX6* contains only motif 1 and motif 2. Moreover, in the Intermediate Clade subfamily both *VdWOX1* and *VdWOX4* contain motif 6. Among the 10 motifs, Motif 1 and Motif 10 comprise 50 amino acids each, whereas Motif 8 and Motif 9 contain 7 and 8 amino acids respectively (Fig. 4C). To some extent, the specificity of the motifs in different subfamilies may lead to functional differences in the *VdWOX* genes. In addition, two pairs of genes (*VdWOX3* and *VdWOX5*, *VdWOX2* and *VdWOX8*) had very high similarity in gene structure and conserved motifs, which indicated that they might have redundant functions in blueberry.

### Three‑dimensional structure analysis of *VdWOX* gene family

**Fig. 5:**
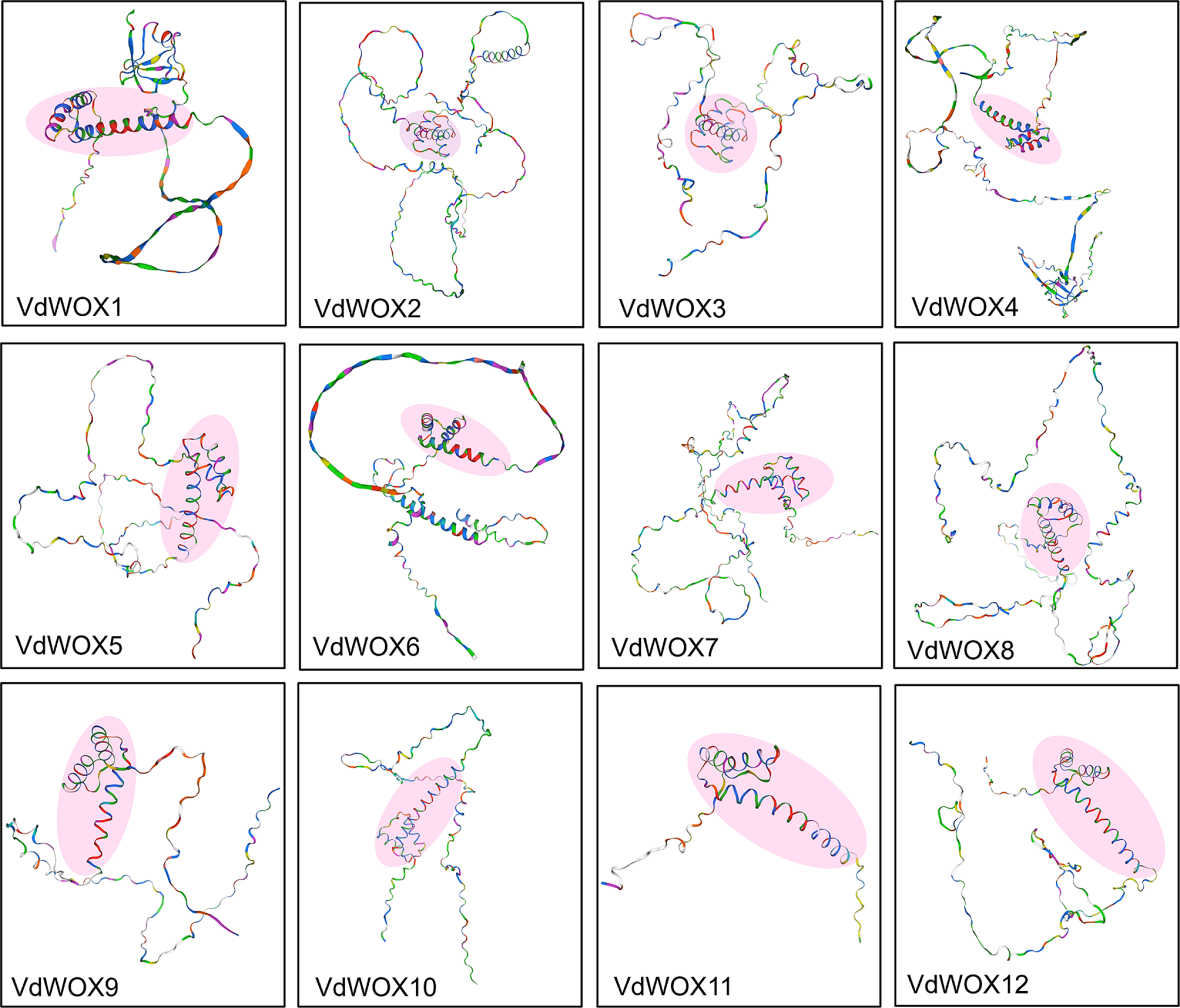
Prediction of the tertiary structure of blueberry WOX family proteins. The conservative homeodomain is colored in pink

In order to understand the structure of VdWOX proteins, a VdWOX proteins model was constructed using AtWOX proteins as a template on SWISS-MODEL. The results showed that although the 12 VdWOX proteins differed in length and morphology, they all contained a conserved homeodomain, which is typical WOX proteins in plants (Fig. 5). The homeodomain was characterised as helix-loop-helix, which was composed of 60–66 amino acid residues. The same observation was obtained from the sequence comparison analysis (Fig. 2C).

### Synteny analysis of the *WOX* gene family

**Fig. 6:**
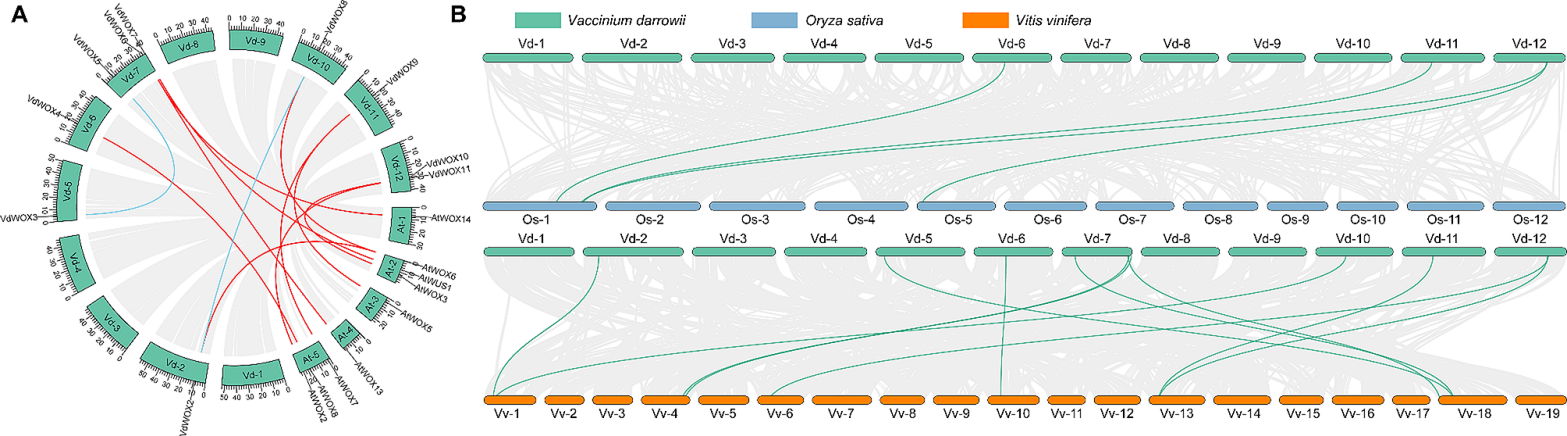
Intraspecific and interspecific collinearity relationships of blueberry *WOX* family genes. (**A**) The blue lines indicated collinearity within the blueberry *WOX* family genes. The red lines indicated the collinearity relationship of the *WOX* family genes between blueberry and *Arabidopsis*. (**B**) Collinearity relationship diagram of *WOX* gene family in blueberry, rice and grape

Gene duplication occurs universally during plant evolution, including whole genome duplication, tandem duplication, segmental duplication and gene duplication can produce homologous genes that share sequence similarities. Synteny analysis was performed to determine the evolutionary characteristics of the *WOX* gene family. Intraspecies collinearity analysis of *VdWOX* gene family and interspecies collinearity analysis with *Arabidopsis* revealed two pairs of fragment duplication events in members of the *VdWOX* family of genes: *VdWOX2* and *VdWOX8*, *VdWOX3* and *VdWOX5* (Fig. 6A). Whereas 10 pairs of genes had collinearity in the *VdWOXs* and *AtWOXs*: *VdWOX2* and *AtWOX6*, *VdWOX4* and *AtWOX8*, *VdWOX6* and *AtWOX13*, *VdWOX7* and *AtWUS1*, *VdWOX7* and *AtWOX14*, *VdWOX8* and *AtWOX6*, *VdWOX9* and *AtWOX5*, *VdWOX9* and *AtWOX7*, *VdWOX10* and *AtWOX3*, *VdWOX10* and *AtWOX2*. In the phylogenetic analysis, *VdWOX7* and *AtWUS1* were in the same branch (Fig. 3), which was consistent with the results of the covariance analysis, suggesting that *VdWOX7* and *AtWUS1* may have similar functions in plant growth and development as well as in stress response.

Simultaneously, rice and grape were also selected for collinearity analysis with blueberry. The results revealed that in the comparison between blueberry and japonica rice, 4 pairs of genes exhibited collinear relationships (Fig. 6B). Similarly, in the comparison between blueberry and grape, 11 pairs of genes were found to have collinear relationships, indicating a high degree of homology between the *VdWOX* genes of blueberry and the *VvWOX* genes of grape, both being woody plants (Fig. 6B), suggesting a close relationship between them. Furthermore, the results indicated that in the collinearity with the genomes of monocotyledonous and dicotyledonous plants, blueberry exhibited one-to-many or many-to-one orthologous genes. These genes underwent multiple duplication events, implying intimate phylogenetic relationships among species. Their evolutionary functions are likely conserved, with their ancestral functions remaining unchanged or unaltered during duplication, thus playing crucial roles in the evolution of the *WOX* gene family.

### *VdWOXs* promoter regions contain the key cis‑elements for phytohormone and stress response

**Fig. 7:**
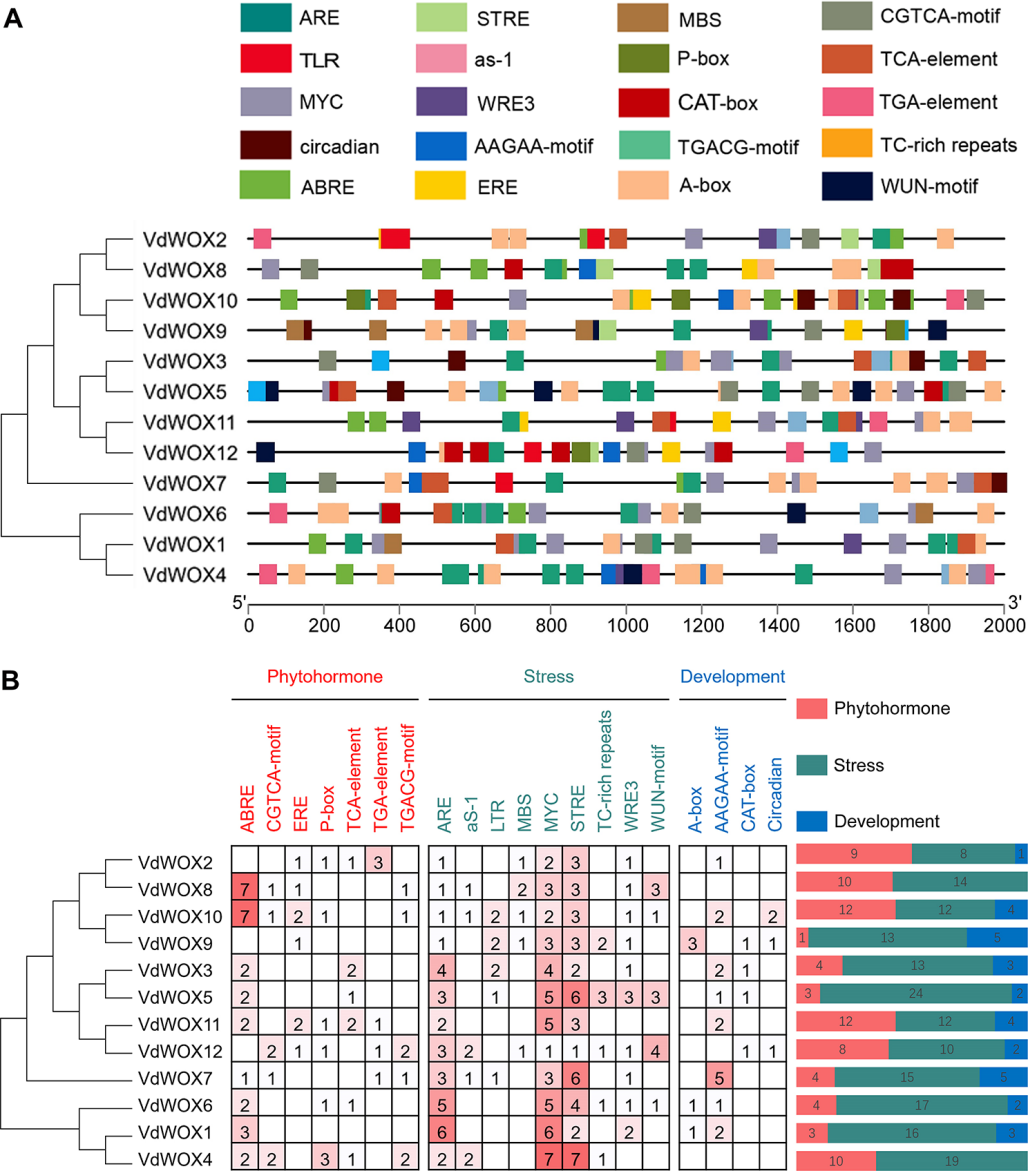
Analysis of Cis-regulatory element in the promoter of *VdWOX* genes. (**A**) Cis-regulatory element; (**B**) Statistics of Cis-regulatory element contained in *VdWOX* family genes

Cis-elements are non-coding DNA sequences in the promoter region of a gene, which are crucial for gene expression and widely involved in the regulation of plant growth, development, and stress response [39]. To better understand the regulatory network of *VdWOX* genes, we analyzed their promoter regions. Further analysis of the promoter regions of the *VdWOX* gene family revealed that they all contain cis-elements associated with plant growth and development, phytohormones and stress responses (Fig. 7A). The response elements identified in the 2000 bp promoter region upstream of the blueberry *WOX* family genes can be classified into three categories based on their functions. Of these, 29 elements were related to plant growth and development, 72 elements were related to phytohormone response pathways and 175 elements were related to abiotic and biotic stresses (Fig. 7B). The most abundant cis-elements in the promoter region of *VdWOX* family genes were salt stress response element (MYC), stress response element (STRE), anaerobic stress-related element (ARE), abscisic acid response element (ABRE) and growth hormone response element (AAGAA-motif), suggesting that the *VdWOX* gene family may be involved in a variety of pathways of growth, development and stress response in blueberry.

### Analysis of *VdWOX* gene family expression patterns

**Fig. 8:**
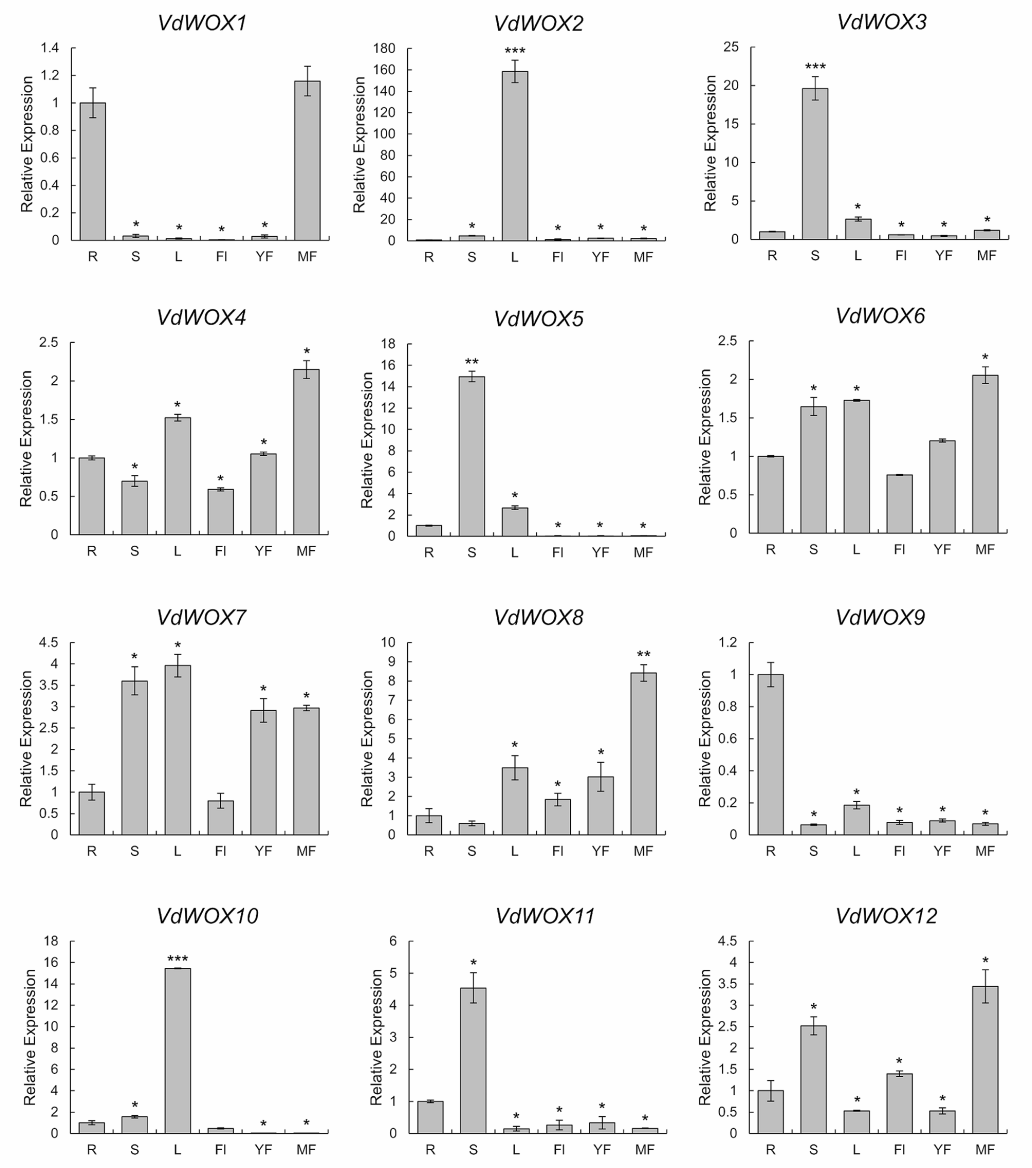
Expression pattern of *VdWOX* genes in blueberry. Roots, stems, leaves, flowers, young and mature fruits were obtained from annual blueberry seedlings. Transcription products of *VdWOXs* in different tissues of blueberry were determined by real-time quantitative PCR. The relative expression of each *VdWOXs* in roots was set normalised to “1”. The blueberry *VdTub2* gene was used as the reference gene. R roots, S stems, L leaves, Fl flowers, YF young fruits, MF mature fruit. Values were means and standard deviations of three biological replicates. Data are presented as the means ± SDs. biological replicates were used for the statistical analyses. Asterisks indicate significant differences (*t* test) compared to. * and *** indicated p-value < 0.05 and < 0.001, respectively

In *Arabidopsis*, *AtWOX* genes were abundant in roots, leaves, stems, flowers and seeds [40]. In rice *OsWOX* were involved in the development of all organs [34]. In paper mulberry, most of the *BpWOX* had relatively high expression in terminal yards and stems, and *BpWOX1* was mainly expressed in terminal buds and leaves [41]. In Citrus sinensis, *CsWUS* was most highly expressed in flowers and stems, while *CsWOX1* and *CsWOX2* were constitutively expressed in all tissues [42]. In this study, we found high expression of *VdWOX* genes in stems, leaves and mature fruits (Fig. 8), which suggested that they played an important role in blueberry plant growth increase and fruit development. In particular, *VdWOX2* had the highest expression in leaves, indicating that *VdWOX2* might be involved in leaf formation and physiological processes.

### *VdWOX* genes expression in response to salt and drought stress in blueberry

Previous studies had shown that *WOX* genes played an important role in regulating plant responses to abiotic stresses [8]. In rice, drought tolerance was enhanced in transgenic overexpressing *OsWOX13* [43]. In poplar, *PagWOX11/12a* responsed to drought stress by promoting root elongation and biomass increase, thereby enhancing drought tolerance in poplar [44, 45]. In apple, *MdWOX13-1* improved drought tolerance by enhancing the ability to scavenge reactive oxygen species [46].

**Fig. 9:**
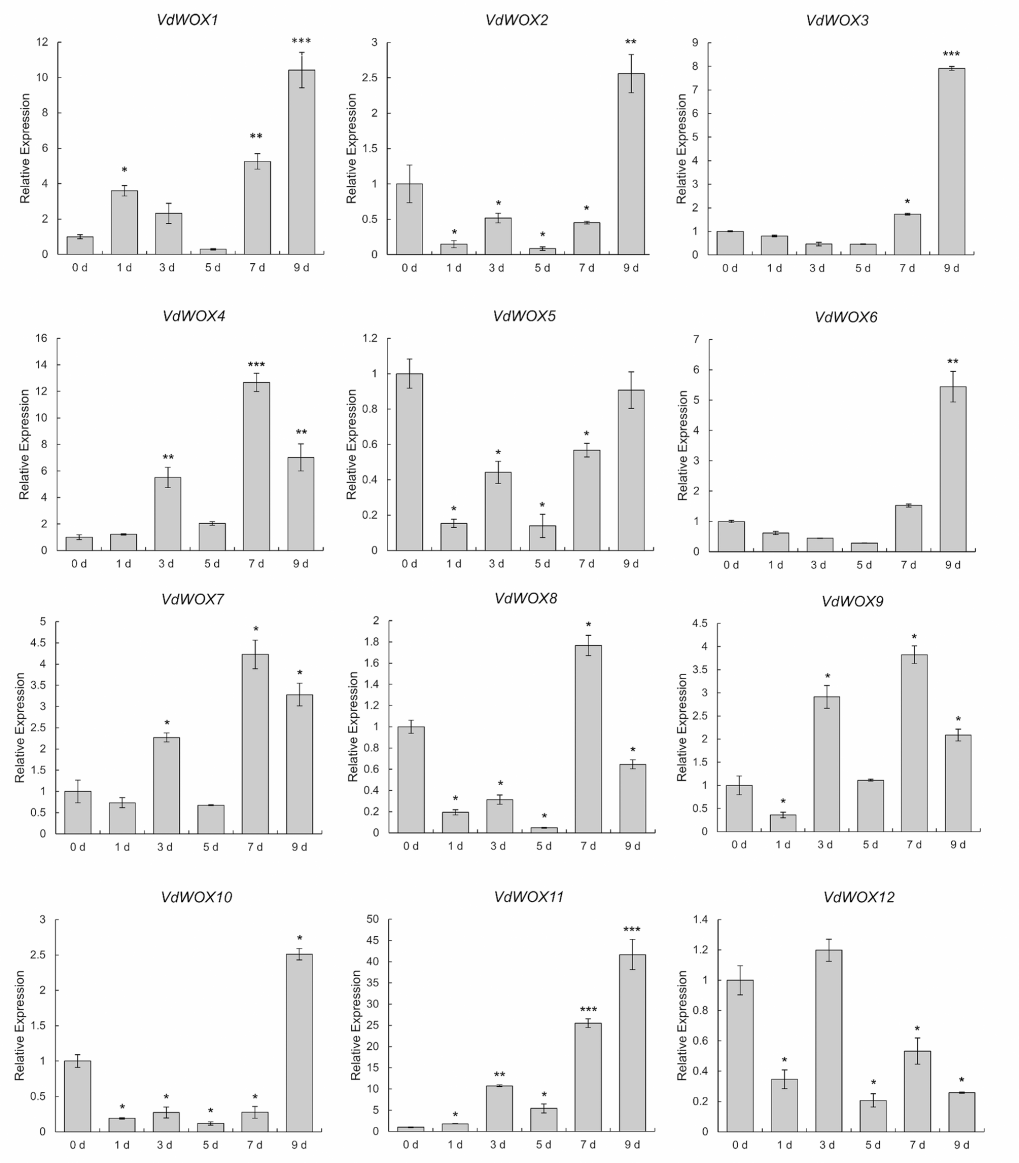
Analysis of the expression pattern of *VdWOX* genes under 200 mM NaCl treatment. The samples were treated for 0 d, 1 d, 3 d, 6 d, 7 d and 9 d. The transcript level of *VdWOXs* at 0 h was set to “1.” Average values and standard deviation of three biological replicates were showed. *, **and*** indicate p-values < 0.05, < 0.01and < 0.001, respectively

To investigate the response of *VdWOX* genes to abiotic stresses, blueberry seedlings were treated with 200 mM NaCl, and samples were taken at 0 d, 1 d, 3 d, 5 d, 7 d and 9 d respectively. Quantitative PCR analysis showed that the expression of all *VdWOX* genes was responsive to salt stress (Fig. 9). The expression of 5 genes (*VdWOX1*, *VdWOX4*, *VdWOX7*, *VdWOX9* and *VdWOX11*) was up-regulated after treatment. The expression of 5 genes (*VdWOX2*, *VdWOX3*, *VdWOX6*, *VdWOX8* and *VdWOX10*) was down-regulated and then up-regulated. And the expression of 2 genes (*VdWOX5* and *VdWOX12*) was down-regulated. Moreover, the expression of 6 genes (*VdWOX1*, *VdWOX2*, *VdWOX3*, *VdWOX6*, *VdWOX10* and *VdWOX11*) reached the highest level at 9 d after treatment.

**Fig. 10:**
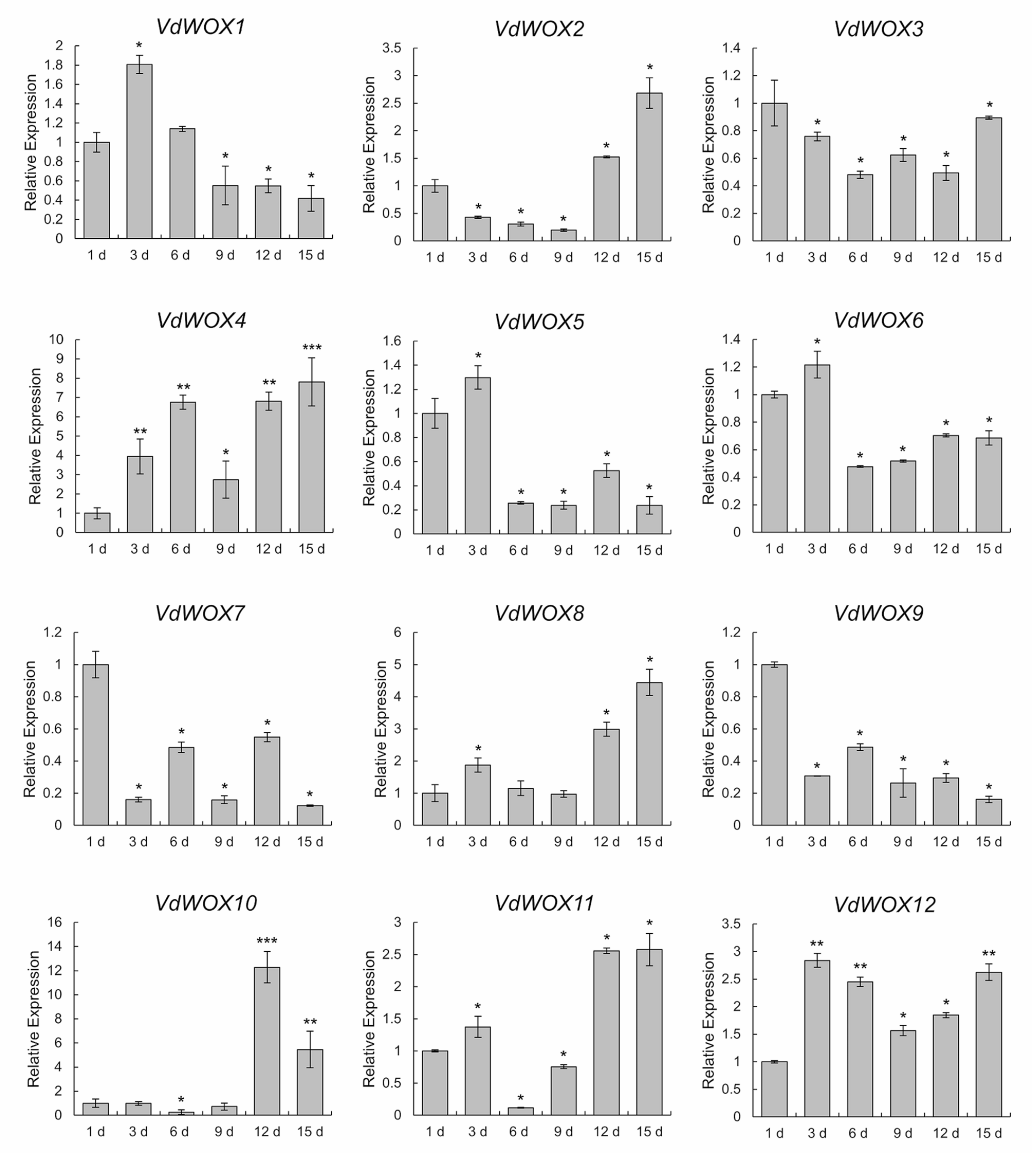
Expression pattern analysis of *VdWOX* genes under drought stress. After 1 d, 3 d, 6 d, 9 d, 12 d and 15 d of drought treatment, the transcription level of *VdWOXs* at 1 d was set to “1”. Average values and standard deviation of three biological replicates were showed. Data are presented as the means ± SDs. biological replicates were used for the statistical analyses. Asterisks indicate significant differences (*t* test) compared to. *, **and*** indicate p-values < 0.05, < 0.01and < 0.001, respectively

Regarding drought treatment, samples were taken at 1 d, 3 d, 6 d, 9 d, 12 d and 15 d of water deficit. Quantitative PCR analysis showed that the expression of all *VdWOX* genes appeared differentially in response to drought stress (Fig. 10). The expression of *VdWOX3*, *VdWOX7* and *VdWOX9* was down-regulated. However, the expression of *VdWOX4*, *VdWOX8*, *VdWOX11* and *VdWOX12* was up-regulated. The expression of *VdWOX2* was first down-regulated and then up-regulated. Moreover, the expression of *VdWOX1*, *VdWOX5*, *VdWOX6* and *VdWOX10* was first up-regulated and then down-regulated.

## Discussion

The *WOX* gene family is one of the highly conserved transcription factor family in plants. Their function is essential for normal plant development and mutations often lead to severe defects. Due to their functional importance, current research on plants is both intensive and extensive. However, to date, studies on the function of *VdWOX* genes were very limited, and the members of the *VdWOX* gene family have not been systematically identified and defined. In this study, we identified 12 different *VdWOX* genes from the blueberry reference genome, which were distributed on eight blueberry chromosomes, and performed bioinformatic analyses of the gene family, including gene structure, conserved motifs, cis-regulatory elements, physicochemical properties of proteins, predicted structures and subcellular localization, which provided a framework for further studies of the gene family.

In this study and reported horticultural crops, the WOX family had been stably divided into three clades (WUS clade, intermediate clade and ancient clade). The two typical conservative domains of the WOX family were HD and WUS-box. In a previous study, the WUS-box domain existed in the WUS clade [3], which was once again confirmed in conservative motif analysis. From the evolutionary relationship of WOX family proteins, the WUS clade was the clade with the highest number of WOX family members, reaching 75%. Each gene in the blueberry WOX family had a synchronous corresponding gene in *Arabidopsis* and grape, indicating that the differentiation of the WOX family preceded the differentiation of the grape, blueberry and *Arabidopsis* species. In terms of evolutionary relationships, grape as a woody plant was more closely related to blueberry. The number of WOX family members in *Arabidopsis*, barley, grape and rice varied on different evolutionary branches, indicating that the WOX branches of each species underwent different evolution after species differentiation (Fig. 3) [23]. As a transcription factor, WOX contains a DNA-binding domain, a trans-regulatory domain, a nuclear localization signal and an oligomerisation site. The DNA-binding domain is the homologous domain of WOXs proteins, and previous studies had shown that *AtWUS1*, *AtWOX3*, *AtWOX4* and *AtWOX11* were localized in the nucleus in *Arabidopsis thaliana* [47]. Moreover, *PtoWUSa, PtoWOX4a*, *PtoWOX5a*, *PtoWOX11/12a* and *PtoWOX13* were also localized in the nucleus in *Populus* [48]. Through subcellular localization prediction, all of the VdWOX proteins were localized in the nucleus in blueberry (Table 1).

The gene structure and motif analysis results indicate that the *WOX* gene family in blueberries has a unique motif, and all genes contain motif 1 and motif 2. Different branches also have their own unique themes, for example, motif 6 only exists in the Intermediate Clade subfamily, while motif 6 only exists in the WUS Clade. Similarly, in Melastoma dodecandrum, AncientClade has motif 3, motif 6 and motif 10 that do not exist in other evolutionary branches. Intermediate Clade has a unique motif 4 [49]. This phenomenon indicates that *WOX* genes in different evolutionary branches may have different functions.

The *WOX* gene family plays an important role in regulating plant growth and development, stress resistance, and plant hormone signal transduction [6, 38]. The promoter of *VdWOX* contains cis acting elements related to plant growth and development, hormones, and stress resistance. Research has shown that the *WOX* genes is regulated by hormones such as CK, IAA and ABA in the regulation of plant growth and development [16, 50]. In Arabidopsis, WUS exhibits a resistive effect and limits the IAA signaling pathway to maintain stem cell characteristics [51]. The CK signal can directly activate the dynamic pattern of WUS expression, and the positive feedback loop between cytokinin signal and WUS function affects the stem meristem tissue pattern [52]. Many hormone responsive elements were also observed in the *VdWOX* promoter region, including ABRE elements, auxin responsive elements, TGA elements, TCA elements, and GARE motifs. Therefore, the *VdWOX* gene family plays a crucial role in plant growth, development, and stress resistance in blueberries by mediating plant hormone regulation.

In *Arabidopsis*, *AtWOX1* and *AtWOX3* play a role in leaf development, especially leaf extension and margin development, and the phenotype of *Arabidopsis* single or double mutants exhibits narrow leaves [53]. In leaves, *VdWOX2* (the closest relative to *AtWOX1*) and *VdWOX10* (the closest relative to *AtWOX3*) exhibited the highest expression levels (Fig. 8). This finding indicated a potential functional redundancy between these 2 genes in leaf development. *AtWOX4* plays a crucial role in facilitating the growth of stem cells in both the procambium and cambium. It is specifically expressed in these two regions [53]. Similarly, *VdWOX3* and *VdWOX10*, which shares similarities with *AtWOX4*, also shows significant expression in the stem. *AtWOX5* in *Arabidopsis* leads to ectopic flower formation and altered root morphology [54]. Meanwhile, *PtoWOX5a* is involved in adventitious root development in poplar [55]. *VdWOX9* is closest to *AtWOX5*, which exhibited the highest expression level in roots, indicating that it functions in roots (Figs. 3 and 8).

Plants grow in a fixed location, but their surrounding environment is perennially changing, so they need to regulate their metabolism to adapt to environmental changes in order to meet their growth needs. Previous studies on *WOX* genes had focused on plant development, and few studies had been conducted on the stress caused by environmental changes. Our results indicated that *VdWOX* genes were indeed responsive to environmental stresses. Salt stress induced the expression of *VdWOX* genes. The expression of *VdWOX2* and *VdWOX10* reached a maximum at 9 days after 200mM NaCl treatment (Fig. 9). Additionally, the expression of *VdWOX2* and *VdWOX10* was found to be highest in leaves (Fig. 8). These findings suggested that blueberries might adapt to high salt environments by altering physiological and biochemical mechanisms of *VdWOX* regulation in leaves. Whereas drought treatment may repress *VdWOX7* expression (Fig. 10). In the phylogenetic tree, *VdWOX7* is the most closely related to *Arabidopsis AtWUS1* (Fig. 3). *VdWOX7* is primarily expressed in terminal buds and leaves (Fig. 8). However, during drought stress, the terminal buds and leaves are susceptible to damage, leading to a reduction in the expression of *VdWOX7*.

In recent years, WOX family members had been successively cloned and characterized in most plants, and the biological functions of some of them had been analyzed. The involvement of WOX in the regulation of plants roots, stems, leaves, flowers, fruits, seeds, embryonic development and abiotic stress had been reported in numerous studies [3, 22, 56–60], which had led to a deeper understanding and appreciation of the role of WOX in the regulation of plant growth and development. These studies had led to a deeper knowledge and understanding of the role of WOX in the regulation of plant growth and development. However, studies on the involvement of WOX in the regulation of plant stress tolerance were still very limited, with relevant studies mainly focusing on plant species such as *Arabidopsis thaliana* [18], rice [43], poplar [44], cotton [61] and little relevant studies on horticultural crops. We used bioinformatics to identify the members of the *VdWOX* gene family, analyzed their gene structures, protein properties, tissue expression and the expression under stress in blueberry. It provides theoretical significance for the future in-depth analysis of the molecular mechanism of the role of *VdWOX* gene family. However, at the same time, our research also has limitations. The focus of our research is to use bioinformatics to identify the members of the *WOX* gene family in blueberries and predict their functions. Further research is needed to clarify the characterization of gene function and explain the mechanism of gene functional expression. At the same time, the excavation of *VdWOX* upstream regulators provides theoretical basis and new ideas for downstream target genes to regulate the plant growth and development processes and response to stress.

## Conclusions

In this study, we identified 12 members of the *WOX* gene family in blueberry. These genes were classified into 3 branches based on evolutionary relationships, WUS Clade, Intermediate Clade and Ancient Clade. The analysis of cis-acting elements showed that the *VdWOX* gene family had many hormone and stress response elements. Through analyzing the expression levels of *VdWOX* genes in different tissues and under salt and drought stress, several *VdWOX* genes were found to be expressed specifically in different tissues and response to various stresses. Among them, *VdWOX2* had the highest expression in leaves, the expression of *VdWOX5* and *VdWOX12* is down-regulated after NaCl treatment, and the expression of *VdWOX3*, *VdWOX7* and *VdWOX9* are up-regulated after drought treatment. These results will contribute to provide a theoretical basis for revealing the molecular mechanism of the *VdWOX* gene family.

### Electronic supplementary material

Below is the link to the electronic supplementary material.

Supplementary Material 1

Supplementary Material 2

Supplementary Material 3

Supplementary Material 4

Supplementary Material 5

## Data Availability

Data will be made available on request. If you require data from this study, please contact: Yanwen Wang, Email: 15684198802@163.com.
